# Quality or equality? The Norwegian experience with medical monopolies

**DOI:** 10.1186/1472-6963-7-20

**Published:** 2007-02-15

**Authors:** Knut Rasmussen, Dag Bratlid

**Affiliations:** 1 Department of Cardiology, University Hospital of North Norway, Norway; 2 Institute of Clinical Medicine, University of Tromsø, 9038 Tromsø, Norway; 3 Department of Pediatrics and Adolescent Medicine, St. Olavs University Hospital, Norway; 4 Institute of Laboratory Medicine, Children's and Women's Diseases, Faculty of Medicine, the Norwegian University for Science and Technology, 7006 Trondheim, Norway

## Abstract

**Background:**

In order to maintain both quality and efficiency of health services in a small country with a scattered population, Norway established a monopoly system for 38 highly specialized medical services. The geographical distributions of these services, which are provided by one or two university hospitals only, were analysed.

**Methods:**

The counties of residence for 2 711 patients admitted for the first time in 2001 to these 31 monopolies and 7 duopolies were identified.

**Results:**

The general tendency observed was that with increasing distance from residential home to monopoly hospitals there was a declining coverage of these health services. The same pattern was found even with regard to explicit diagnoses or treatments such as organ transplantations (except renal transplantations). Duopolies seemed to yield a more even geographical distribution of the services.

**Conclusion:**

Monopolies may serve as a useful means for maintaining quality in highly specialized medical services, but seem to have an inherent tendency to do this at the expense of geographical equality.

## Background

During the last 50 years a constant movement of decentralization has taken place in Norwegian health care. One of the driving forces of this movement has been the documentation of a poorer health status and a poorer health service in many parts of district Norway. The University of Tromsø was thus established primarily in order to educate doctors and bring specialized health service to the region. Five health regions, each with a University Hospital, have later emerged as the main providers and coordinators of highly specialized health care.

During this process increasing concerns have been raised regarding the ability of many small hospitals to maintain quality and efficiency for procedures regarded as the most difficult, costly and critical. Therefore, in 1990, a monopoly system was established, defining a number of highly specialized and costly medical services in which there was both a duty for county hospitals to refer patients as well as a duty for the designated university hospitals to admit patients in these groups.

By means of control of purchasing of costly equipment as well as establishing of special laboratory services and personnel at selected university hospitals, the government ascertained that treatment of patients with rare or complicated diseases were not split between several hospitals, when each of which would not reach a sufficient patient volume to secure adequate quality of treatment. An important issue was also to secure that national investments in expensive medical equipment as well as resources for training of highly qualified personnel were targeted to meet the needs on a national level, rather than to the need of individual hospitals to offer patients a complete set of the most modern and expensive equipments and treatments.

An analysis of the performance of this unique monopoly system may offer some lesson also for other countries trying to maintain a balance between too much and too little centralization in relation to quality and cost control. We hereby report on what we believe is a fundamental dilemma facing monopoly systems in health care.

### The system

In order to advise the health authorities on the structure and function of these national monopoly services a professional advisory board was established. Each service was followed and evaluated through regular reports regarding efficiency, quality, geographical distribution and economy as well as by site visits. During the years new monopoly functions have been established while others have been deregulated and transferred either to duopolies or to regionalized functions to be provided by all university hospitals. Each service should also have a national steering or advisory committee.

By 2001 there were 31 monopoly functions, 24 of which were located at the two University Hospitals in the capital of Oslo (The National Hospital, regional Hospital of Health Region South, and Ullevål University Hospital, the regional hospital of Health Region East), four were located in Bergen and three in Trondheim. Seven duopolies were split between the same hospitals. None of the functions were located in Northern Norway. The monopolies included such services as all types of organ transplantation, cochlea implants in children, surgical treatment of epilepsy, retinoblastoma, advanced prenatal surgical procedures and treatment of severe burn injuries. The duopolies included surgery for congenital heart disease in children and neonatal surgery. Monopolies admitted from 0 to 431 new patients every year, duopolies from 0 to 200 new patients at each hospital (Table 1).

**Table 1:** Patient volume in 2001 (patients admitted for the first time) for highly specialized medical services centralized to one (monopolies) or two (bipolies) university hospitals in Norway.

**Services and/or diagnoses allocated to health service monopolies or duopolies**	**Volume hospital 1**	**Volume hospital 2**	**Location (city) of service**
All organ transplantations#	354		Oslo
Cardiac arrhytmias (surgical treatment)	5		Oslo
Rheuma surgery in children	53		Oslo
Elective surgery in haemophiliacs	37		Oslo
Cochlea implants in children	33		Oslo
Advanced craniofascial surgery	74		Oslo
Hypoplastic left heart syndrome	8		Oslo
Embolization of cerebral A-V malformations	46		Oslo
Phenylketonuria	5		Oslo
Advanced replantation surgery	48		Oslo
Advanced retinal surgery	0		Oslo
Retinoblastoma	3		Oslo
Trans-sexualism	6		Oslo
Large hemangiomas and vascular malformations	153		Oslo
Diagnosis and treatment of severe epilepsia*	431		Oslo
Surgical treatment of epilepsia	50		Oslo
Choriocarcinoma	4		Oslo
Exentration surgery for gynaecologic. cancer	5		Oslo
Perfusion chemotherapy	5		Oslo
Congenital glaucoma	11		Oslo
Oculoplasty	71		Oslo
Advanced burns	70		Bergen
Elective hyperbar treatment	251		Bergen
Treatment with keratoprosthesis	0		Bergen
Radiation (knife) neurosurgery	178		Bergen
Photopheresis	5		Trondheim
Advanced invasive foetal care	71		Trondheim
Complicated neck and spine disorders	8		Trondheim
Neonatal surgery (excluded cardiac)	80	38	Oslo/Trondheim
Sperm bank	107	94	Oslo/Trondheim
Elective open heart surgery in children	200	49	Oslo/Bergen
Cochlea implants in adults	21	6	Oslo/Bergen
Intersex	8	11	Oslo/Bergen
Cleft lip-jaw-palate	113	45	Oslo/Bergen
Brachytherapy	0	4	Oslo/Bergen

#Includes heart, lung, liver, pancreas, renal and allogenic bone marrow transplantations.

*Includes also the services habilitation for severe epilepsia and pre-surgical evaluation for surgical treatment of epilepsia.

## Methods

In 2001 a thorough review of the entire system was performed [1]. The review was designed to answer the question if this centralized system, in addition to securing services of adequate quality, also was equally accessible for patients throughout the country. The review included the identification of the counties of residence for each of the 2 711 patients admitted and treated for the first time that year. For the present purpose, the total number of patients admitted in these services in relation to county of residence, patient volume and residence in strict monopolies as well as patient volume and residence for non-renal transplantations particularly, are presented. Since all studied patient data were non-identifiable group data, the study was exempt from the need of ethical approval.

For analysis of distribution of services the patient volume from the three northernmost counties (population 464 000) are compared with the remaining 16 counties (population 4 058 000). Furthermore, the combined three northernmost counties and the four counties in the central and west part of the country ("District Norway", population 1 208 000) are compared with the remaining 12 southern counties (population 3 314 000).

### Statistical analysis

Statistical analysis was performed by calculating odds ratio (OR) and confidence intervals for the possibility of being admitted to any of the centralized highly specialized services for a resident of the northern counties, as well as "district Norway" compared to residents of the rest of the country.

## Results

Table 2 gives the odds ratios and confidence intervals for the possibility of being admitted to any of the centralized highly specialized services for a resident of the three northern counties, as well as "district Norway" compared to residents of the remaining 16 and 12 counties in the rest of the country, respectively. As indicated, the general tendency is that people living in the north and in "district Norway" have a substantially reduced chance of being admitted to these highly specialized services. When only the 31 monopoly functions are analyzed the odds ratios are somewhat smaller than for the all services combined. For non-renal organ transplantation the chances of having access to treatment for a resident in the north is about 1/3 as for residents in the rest of the country. All the differences are statistically highly significant with p-levels below 0.001, except for the comparison of the northern counties versus the rest with regard to organ transplantations, which has a p-level of 0.007. To illustrate the variation between different counties, data for organ transplantation, radiation neurosurgery and advanced invasive foetal care, are given in Table 3.

**Table 2:** Odds ratio and confidence intervals for the possibility for patients from the northern counties of Norway (North) as well as the northern and central-west counties combined ("District Norway") to be admitted in one of the 38 centralized highly specialized services compared with patients from the rest of the country (Rest) in 2001.

	**OR**	**95 % CI**
**Total services (monopolies and duopolies)**		
North versus Rest	0.77	0.67 – 0.89
"District Norway" versus Rest	0.78	0.71 – 0.86
**Monopolies**		
North versus Rest	0.73	0.62 – 0.86
"District Norway" versus Rest	0.66	0.59 – 0.73
**Non Renal Tx**		
North versus Rest	0.28	0.09 – 0.78
"District Norway" versus Rest	0.33	0.18 – 0.60

**Table 3:** Examples of uneven geographical distribution (incidence for treatment) of patients from the different 19 Norwegian counties to the monopoly functions of organ transplantation (located at in Oslo, the Univeristy Hospital of Health Region South), radiation neurosurgery (located in Bergen, Health Region West) and Advanced invasive foetal care (located in Trondheim, Health Region Middle) treated in 2001. Incidence for treatment is given as treated patients pr. 100.000 residents.

**Health Region and County of residence**	**Organ transplantation**	**Radiation neurosurgery**	**Advanced invasive foetal care**
**Health Region East**	8.7	2.9	0.7
Østfold	11.5	2.0	0
Hedmark	5.3	2.1	4.8
Oppland	10.4	1.6	1.1
Oslo	6.6	3.3	0
Akershus	10.9	3.6	0
**Health Region South**	8.9	2.3	0
Buskerud	10.0	2.1	0
Vestfold	11.6	0.9	0
Telemark	9.0	0.6	0
Aust-Agder	5.3	4.9	0
Vest-Agder	5.1	4.5	0
**Health Region West**	7.6	9.2	1.9
Rogaland	7.4	7.9	4.7
Hordaland	8.5	10.1	0
Sogn og Fjordane	4.7	10.3	0
**Health Region Middle**	5.2	3.0	4.4
Møre og Romsdal	2.9	2.9	4.5
Sør-Trøndelag	4.9	4.5	4.5
Nord-Trøndelag	10.2	0	3.9
**Health Region North**	5.8	1.8	3.0
Nordland	6.7	2.5	5.0
Troms	5.3	0.7	0.7
Finnmark	4.1	1.4	1.4
**National average**	7.9	3,9	1.6

**Figure 1:**
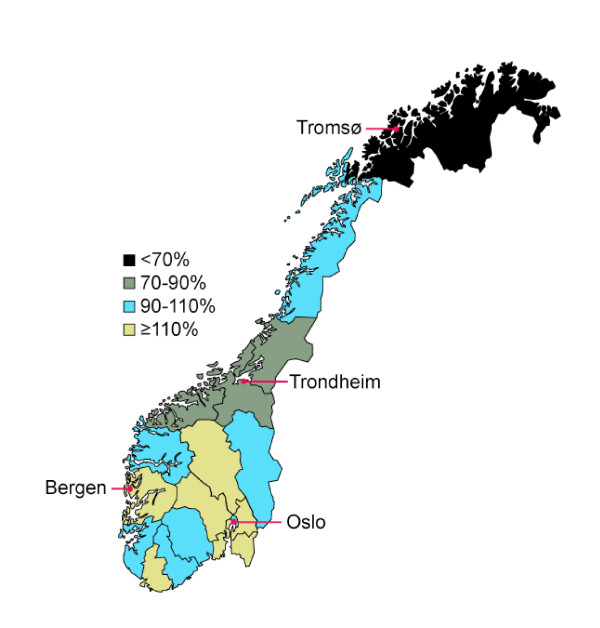
Variations between Norwegian counties in usage (admitted patients/100 000 residents) of 38 highly specialized and centralized medical services. County usage values are given as the percentage of the national average. The monopoly/duopoly services are located at the university hospitals in Oslo, Bergen and Trondheim, while the University hospital in Tromsø does not have any such clinical functions.

Figure 1 summarizes the geographical distribution of the total volume of highly specialized services in all 19 counties.

## Discussions and conclusion

The significance of the results of the present study depends on a complete and correct patient volume being reported for the different services. As pointed out earlier, by government control, only the designated hospital would in Norway have access to both the necessary technology and qualified personnel needed to perform the defined treatment (Table 1). If a patient at a local hospital was found to be in need of any of these services, the patient would thus either be remitted to the designated hospital for treatment, or not given the treatment at all. Only isolated cases have been referred to hospitals out of the country, and then usually after being remitted by the monopoly hospital. It should also be noted that in Norway all institutional health care, including highly specialized services, is provided free of charge for all citizens. The patient volumes analyzed for these strictly defined services are therefore as complete as possible and based on all available national health statistics.

Some of the monopoly or duopoly functions are, however, not precisely defined, neither medically or technically. For such, where the centralized care is defined for the "advanced", "large" or "complicated" stages of disease, patient selection for remittance would be dependant on the medical evaluation and the level of care at the local hospital. Variations in the incidence of patients being remitted by the different counties might for such conditions therefore reflect either a real variation in actual remittance (we will treat them ourselves), or a variation in the definition of which patients would need these services (this is not advanced invasive foetal care). Analyses of variations in case-mix and mortality patterns between different counties would not be of any help in evaluating this, since such differences in disease patterns (advanced, large, complicated) are not reflected in the national statistics of diagnoses or procedures.

The management system for the centralized, highly specialized medical services in Norway seems in general to have fulfilled the defined purposes, namely to obtain a reasonable quality, efficiency and economy. Although the documentation of quality may be difficult in small patient series, there has definitely been no "Bristol cases" [2] in this patient population.

The data presented indicate, however, that the fulfilling of the original objects has been achieved at the expense of a loss of equal access for all residents. Despite the fact that the performance of these services has been monitored, highly significant differences in access to the services for patients from different parts of the country has been disclosed. This inequality of access is particularly disturbing since the medical conditions and treatments covered, such as organ transplantation, are among the most severe and critical in relation to life or death, and are services defined as having a high medical and political priority in the Norwegian National Health Service [3]. It seems unlikely that the findings can be explained by a lower true demand in the northern and peripheral parts of the country. Most health statistics point in the opposite directions regarding all main disease groups, particularly in the northernmost counties. Thus, the data have probably disclosed another example of "the inverse care law" [4].

A number of explanations for these problems of distribution may be found. The monopoly providers may have difficulties in announcing their services properly to hospitals 2000 km away from the designated centres, and the referral institutions in the peripheral parts of the country may not be properly updated regarding treatment possibilities for small patient groups and niche services. Most importantly, however, it seems that monopoly institutions as such may have an inherent tendency to cause uneven geographical distributions. It may be postulated that monopolies in health care, as in economy, tend to pacify both those in charge of and those outside the monopoly institutions. Some degree of competition may be fruitful, even in medicine.

International debate regarding equal access to health services has in most countries focused on equality linked to race, social factors, gender, education and economy [5-8]. In the Nordic countries and other countries with a scattered population, geographical maldistribution has been the main focus [9]. Such a focus seems to be substantiated by the results of the present study. However, the geographical differences disclosed may to some extent also be interpreted in terms of race and social factors. The counties where patients have the lowest access are thus characterized not only by distance, but also by a lower educational level and a greater ethnic heterogeneity. This interpretation is also supported by the fact that residents of the capital of Oslo, the location of the majority of these monopoly or duopoly services (Table 1), has a lower use of these specialized services both compared to the national average and compared to the more affluent, better educated and ethnical more homogenous residents in the surrounding counties (Fig. 1).

The general experience in Norway is nevertheless that equal access to most health care has developed as a function of our regionalized health care system, where university hospitals were responsible for patient care in five defined regions, Health Region East (population 1.6 million), Helath Region South (population 875.000), Health Region West (population 926.000), Health Region Middle Norway (population 637.000), and Health Region North (population 464.000). In Northern Norway improvements came gradually from 1972 with the foundation of the University Hospital of North Norway and medical school in Tromsø. It has been shown that graduates from this university to a greater extent than graduates from the southern medical schools continue to work in the north, particularly if they are otherwise connected to the region. As a result of this, more medical specialists and more specialized medicine have gradually also became locally available to the people in Northern Norway. In this period unfavourable mortality differences in Northern Norway for most of the major malignant diseases as well as procedure rate differences for invasive cardiology and heart surgery have all been levelled out, and have in some instances even been reversed. In spite of this favourable development, however, there is still an excess total mortality and morbidity in Northern Norway.

Thus, while the regionalized system seems to be adequate for maintaining the balance between quality and equality for the majority of patients and health services in general, including the peripheral parts of the country, the highly specialized monopoly services has not yet reached this level of development. In a regionalized system quality may not always be perfect, but the loss in this aspect is for the patient probably more than retrieved by a gain in accessibility.

Our advice to the health authorities in Norway is therefore that many of these highly specialized services, particularly those with a relatively large patient volume, also could be regionalized, even if this would increase costs. Those who are maintained should be observed and evaluated rigorously, not only with regard to quality, but also to their geographical and social distribution. By the same logic, namely a presumed link between quality and quantity, it could also be argued that some services would be even better off, maybe also in relation to geographical distribution, if they were exported to or coordinated with similar services in other (and larger) countries. This has been suggested but not realized, mainly due to formal issues. The current development of health care within the European Union may facilitate such a development.

The ideal of equality has been vigorously debated both on a philosophical and social level [10,11]. In health care it has often been presupposed that a society with equal access must pay for this in terms of a reduced efficiency and quality [12,13]. This may, however, not be so. In the parts of society with poor access, patients may be found with a high potential for benefiting from the treatment. The ideal of equality may therefore help us optimizing health care for the entire population.

In parallel with increasing affluence as well as level of medical services, it seems that a gradual weakening of the ideal of equality has occurred [14]. The present data may serve to indicate that this ideal needs to be maintained. Even in a Nordic welfare state an even distribution of health care can not be taken for granted. A public, national health care system should be able to give the same quality and level of services to the entire population.

## Competing interests

The author(s) declare that they have no competing interests.

## Authors' contributions

KR and DB have equally contributed in the data collection, analysis and writing of the manuscript. Both authors have read and approved the final manuscript

## Pre-publication history

The pre-publication history for this paper can be accessed here:


